# Evaluation of growth, antioxidant status, hepatic enzymes and immunity of Nanoselenium-Fed *Cirrhinus mrigala*

**DOI:** 10.1371/journal.pone.0308761

**Published:** 2024-08-12

**Authors:** Sobia Nisa, Mahroze Fatima, Syed Zakir Hussain Shah, Noor Khan, Beenish Aftab, Wazir Ali, Maryam, Saba Sana, Amber Fatima

**Affiliations:** 1 Department of Fisheries and Aquaculture, University of Veterinary and Animal Sciences, Lahore, Pakistan; 2 Department of Zoology, University of Gujrat, Gujrat, Pakistan; 3 Institute of Zoology, University of the Punjab, Lahore, Pakistan; 4 Department of Biological Sciences, University of Veterinary & Animal Sciences, Lahore, Pakistan; 5 Institute of Microbiology, University of Veterinary and Animal Sciences, Lahore, Pakistan; Sher-e-Kashmir University of Agricultural Sciences and Technology of Kashmir, INDIA

## Abstract

This study was conducted to investigate the effects of selenium nanoparticle (Se-NP) supplementation on the growth performance, carcass composition, antioxidant status, hepatic enzyme activities, and immunity of *Cirrhinus mrigala*. For this purpose, fish with an average initial weight of 7.44 ± 0.04 g were fed five experimental diets containing 0 (control), 0.25, 0.5, 1, and 2 mg kg^-1^ Se-NPs diets for 90 days. The analysed selenium (Se) contents of the diets were 0.35, 0.64, 0.92, 1.43, and 2.39 mg kg^-1^. Twenty five fish were randomly distributed in each of 5 aquarium (36 × 23.7 × 24.3 inches) in triplicate. The results showed that supplementation with Se up to 0.92 mg/kg significantly increased (p<0.05) weight gain, weight gain% (WG%), and specific growth rate (SGR) by 34%, 33%, and 16%, respectively, compared to the control diet. Dietary Se concentrations up to 0.92 mg/kg significantly increased the crude protein and crude fat and reduced (p<0.05) the moisture content as compared to the control group. Fish fed 0.92 mg kg^-1^ Se had significantly lower malondialdehyde (MDA) contents and higher activities of catalase, superoxide dismutase, and glutathione peroxidase in liver and serum as compared to other experimental diets. Moreover, a significant increase (p<0.05) in the level of serum immunoglobulin and lysozyme (LYZ) activity was recorded in fish fed 0.92 mg/kg Se diet. Moreover, the highest (p<0.05) values of aspartate transaminase (AST) and alanine transaminase (ALT) were recorded in fish fed 2.39 mg/kg Se level. However, serum alkaline phosphatase (ALP) activity remained unaffected by dietary treatment. Broken-line regression analysis indicated that 0.83 mg/kg Se is required for the optimum growth performance of *C*. *mrigala*.

## Introduction

Aquaculture has immense importance in providing the global population with high-quality proteins and future food security. The growing population has exerted great pressure on aquaculture and aquaculture practices have moved towards intensification to meet the demand [1]. However, culturing fish at a high stocking density results in frequent collisions, competition for food and space, and deterioration of water quality. These factors contribute to stress conditions that negatively correlate with the growth and health status of fish [2]. Oxidative stress increases the generation of free radicals, which damages the membranes of cells and leads to depressed growth, immunity, and ultimately mortality. The sustainability of aquaculture has been linked with strategies to reduce stress conditions to boost production and profitability [3]. Several strategies, such as antibiotics, vaccines, and other synthetic chemicals, are being employed, which poses serious health concerns [4]. Dietary manipulation with micronutrients has been suggested as the most effective and safest method to enhance the health status and growth of fish [5].

Selenium is an indispensable trace element required in small quantity that acts as a great immune stimulator and antioxidant [6]. In addition to nutritional requirement, Se is also required for the synthesis of selenoproteins such as glutathione peroxidase (GPx), which is an important antioxidant enzyme that acts as the first line of defense against free radicals [7]. However, selenium acts as both an immune stimulator and toxic with a minute difference in dose. According to a report, 0.2–12 mg kg^-1^ Se is required to supplement the diet of different fish species [8]. Deficiency of Se caused muscular dystrophy, swollen endoplasmic reticulum, and myocardial degeneration in fish [9]. However, high Se levels induced toxic effects on fish health and resulted in teratogenic deformities of fish organs, tissue destruction, nephrocalcinosis, and stunted growth [10]. Moreover, the chemical form of dietary-supplemented Se in feed formulations is an important consideration [10]. Previous studies have shown that organic forms of Se have a better ability to accumulate and be digested in fish bodies than inactive forms [11]. However, in some cases, an organic form of Se has less functionality due to its high pH, which in turn reduces its solubility in water [12].

Nanotechnology is emerging as a novel and safe approach to enhance nutrient absorption and utilization due to the very small size of particles. It has been confirmed that dietary Se nanoparticle (Se-NP) supplementation is more efficient in improving the immune response of fish than organic or inorganic forms [13]. These Se-NPs have gained attention in aquafeeds due to their low toxicity, higher bioactivity, biocompatibility, strong adsorption properties, and high chemical consistency [14]. In crucian carp, supplementation with selenium nanoparticles (Se-NPs) had more positive impacts on the muscle Se content than the organic form [15]. Previous studies have also shown that supplementation with Se-NPs improved the growth performance of different fish species [7,16,17].

*Cirrhinus mrigala* (mori) is an important fish of the Indian subcontinent. It contributes 67% of total freshwater aquaculture production with *Catla catla* and *Labeo rohita* [18]. This species is considered a potential candidate for the polyculture system in the Indo-Pak region. Hence, the formation of nutritionally balanced feed is an absolute need of the moment for intensive farming practices of this species. Therefore, this study was undertaken to determine the optimum level of Se for Cirrhinus mrigala juveniles.

## Materials and methods

### Ethical approval

This study was conducted after approval (No. DR/237) from the Ethical Review Committee of the University of Veterinary and Animal Sciences, Lahore, according to the standard guidelines of the institute.

### Diet formulation

Se-NPs (average particle size: <80 nm; purity>99%; Nanochemazone, Canada) were used as a source of Se. Five experimental diets were formulated containing 0, 0.25, 0.5, 1, and 2 mg/kg Se-NPs (Table 1). Feed ingredients were air-dried, crushed (KENWOOD, AT284), screened (0.05 mm), and thoroughly mixed with Se-NPs and fish oil. Distilled water was used for the formation of stiff dough, which was pelleted through meat mincer (ANEX, AG3060). Pellets (1 mm) were shade-dried and stored in airtight labelled containers for the feeding trial.

**Table 1:** Formulation of experimental diets for *Cirrhinus mrigala* with their chemical composition.

**Ingredients (%)**	**Se concentrations in diets**				
	**0.35**	**0.64**	**0.92**	**1.43**	**2.39**
Soybean meal^a^^a1^	25.00	25.00	25.00	25.00	25.00
Fish meal^b^	10.00	10.00	10.00	10.00	10.00
Sunflower meal^c^	30.00	30.00	30.00	30.00	30.00
Wheat flour^d^	11.00	11.00	11.00	11.00	11.00
Rice polish^e^	15.00	15.00	15.00	15.00	15.00
Fish oil	6.00	6.00	6.00	6.00	6.00
Vitamin premix*	1.00	1.00	1.00	1.00	1.00
Mineral mixture**	1.00	1.00	1.00	1.00	1.00
L-Lysine	0.50	0.50	0.50	0.50	0.50
DL-Methionine	0.50	0.50	0.50	0.50	0.50
Se-NPs (mg/kg)	0	0.25	0.50	1.00	2.00
**Proximate composition (%)**					
Moisture	90.77±0.41	90.82±0.55	90.84±0.44	90.87±0.53	90.79±0.49
Crude protein	31.31±0.23	31.25±0.12	31.28±0.21	31.29±0.19	31.21±0.19
Crude fat	10.31±0.04	10.36±0.02	10.31±0.03	10.35±0.03	10.33±0.04
Crude ash	8.05±0.03	8.09±0.02	8.07±0.04	8.08±0.02	8.09±0.04
Analyzed Se (mg/kg)	0.35±0.03	0.65±0.03	0.92±0.02	1.43±0.03	2.39±0.02

a crude protein 44.09% and crude fat 13.35%.

b crude protein 56.16% and crude fat 10.30%.

c crude protein 40.03 and crude fat 12.05%.

d crude protein 12.14% and crude fat 2%.

e crude protein 11.12% and crude fat 13.54%.

*Vitamin premix (kg^**-1**^ **of diet contain)** Vitamin A (15 M.I.U.), Vitamin D_3_ (3 M.I.U.), Vitamin E (6000 IU), Nicotinic acid (25000 mg), Vitamin B_1_ (5000 mg), Vitamin B_2_ (6000 mg), Vitamin K_3_ (4000 mg), Vitamin B_6_ (4000 mg), Folic acid (750 mg), Vitamin B_12_ (9000 mg), Vitamin C (15000 mg), Calcium pantothenate (10,000 mg).

**Mineral mixture (per gram contains) KH_2_PO_4_ (479 mg g^-1^), MgSO_4_.7H_2_O (153 mg g^-1^), NaCl (15 mg g^-1^), COCl.6H_2_O (0.0816 mg g^-1^), AlCl_3_.6H_2_O (0.255 mg g^-1^), CuSO_4_.5H_2_O (210.67 mg g^-1^), FeSO_4_.H_2_O (100.6 mg g^-1^), MnSO_4_.5H_2_O (116.67 mg g^-1^), ZnSO_4_.7H_2_O (121.33 mg g^-1^), CaCO_3_ (316 mg g^-1^).

### Experimental fish and feeding trial

A total of 375 *C*. *mrigala* juveniles were procured from Arain Fish Farm (local farm located 10 km from laboratory) in Pattoki, Pakistan. Fish were transported in polyethlene bags following the standard protocol described by Berka [19]. Upon arrival in the laboratory, fish were bathed in anesthetic solution (5 g/L KMnO_4_) to remove any parasites. Then, fish were acclimatized in a circular tank (2000 L capacity) with a 0.3 L/min water exchange rate and continuous aeration for 15 day [20]. Fish were fed a control diet twice daily @3% body weight ratio during acclimatization. At the beginning of the experiment, the initial body weight (7.44 ± 0.04 g) of the fish was recorded, and the fish were randomly distributed into 15 aquaria (36 × 23.7 × 24.3 inches) at a density of 25 fish per aquarium. The experimental diets were given to fish @3% body weight ratio twice daily for 90 days. After 3 hours of feeding, uneaten feed was collected from each tank to estimate feed intake (FI). Fish were batch weighed every fortnight, and feeding amount was adjusted accordingly. The water quality was maintained by replacing 50% of the tank water with fresh water daily [21].

The water quality parameters, such as dissolved oxygen, pH, and temperature, were monitored regularly throughout the feeding trial, and their mean values were 7.6 ± 0.3 mg per liter, 7.2 ± 0.2 and 28.5 ± 0.7°C, respectively.

### Sample collection

At the end of 90 days of feeding trial, fish were deprived of feed for 24 hours and then fish were anaesthetized with 25 mg/L tricaine methanesulfonate [22]. For proximate composition analysis, five fish were taken at random from each replicate. Another 5 fish were taken from each replicate and were dissected to collect liver samples for the analysis of antioxidant enzymes activitiesand lipid peroxidation assays. Blood samples were collected from 5 fish of each replicates by puncture the caudal vein using 3 mL sterile syringe. Blood samples were immediately transported in laboratory for serum separation.

### Growth performance and feed utilization

At experiment termination, fish were individually weighed to determine the weight gain (WG), specific growth rate (SGR), feed intake (FI), feed conversion ratio (FCR), and survival rate (SR) using the following standard equations.


Weightgain=finalbodyweight−intialbodyweight

$$Weight\;gain\;\%=\frac{final\;weight-intial\;weight}{intial\;weight\;}\times 100$$

$$SGR=\frac{\ln\left(average\;final\;weight\right)-\ln\left(average\;intial\;weight\right)}{number\;of\;days}\times 100$$

FI(g/day)=feedgiven−unconsumedfeed

$$FCR=\frac{total\;dry\;feed\;intake(g)}{wet\;weight\;gain(g)}$$

$$SR\;\%=\frac{final\;fish\;numbers}{intial\;fish\;numbers}\times 100$$

### Proximate analysis

The AOAC [23] procedure was used for the estimation of proximate composition. Briefly, moisture was determined through the oven-drying method, and crude protein (N×6.25) was estimated by Kjeldahl’s method. The Soxhlet method was used for the determination of crude fat. Crude ash was estimated by placing the samples at 660°C in a muffle furnace. Atomic absorption spectroscopy (Hitachi ZA3000, Chiyoda, Tokyo, Japan) was used for the estimation of Se in diets [24].

### Antioxidant enzyme activities and lipid peroxidation

Hepatic samples from each replicate were pooled and homogenized with phosphate buffer to obtain the extract. After centrifugation of the homogenized samples, the supernatant layer was separated and stored for antioxidant enzyme assays. Glutathione peroxidase (GPx) activity was determined at A_470_ nm by measuring its ability to decrease H_2_O_2_ concentration [25]. Catalase (CAT) activity was estimated by its potential to reduce H_2_O_2_ concentration at 240 nm [26]. The activity of superoxide dismutase (SOD) was estimated by its capacity to prevent photoreduction of nitroblue tetrazole [27]. Malondialdehyde (MDA) content was measured colorimetrically by following Gatta, Pirini [28]. Serum antioxidant enzyme activities and MDA contents were also determined using same methods.

### Immune parameters

Lysozyme (LYZ) activity was estimated by the lysis of *Staphylococcus aureus* by following the standard protocol of Ellis [29]. Total immunoglobulin was determined by following the procedure of [30].


TotalImmunoglobulin=totalptoteinfromsample−proteincontentinsupernatant

### Serum hepatic enzymes

The commercial kit method (CHEMELEX, S. A, Pol. Ind. Can Castells, Spain) was used to determine the activities of hepatic enzymes, including alanine aminotransferase (ALT), alkaline phosphatase (ALP) and aspartate aminotransferase (AST), in serum.

### Statistical analysis

The obtained data were subjected to one-way analysis of variance, and Duncan’s multiple range test was applied to compare the means in SPSS (version 23). To determine the optimum dietary requirement of Se, broken-line regression analysis was used.

## Results

### Growth performance and feed utilization

**Fig 1:**
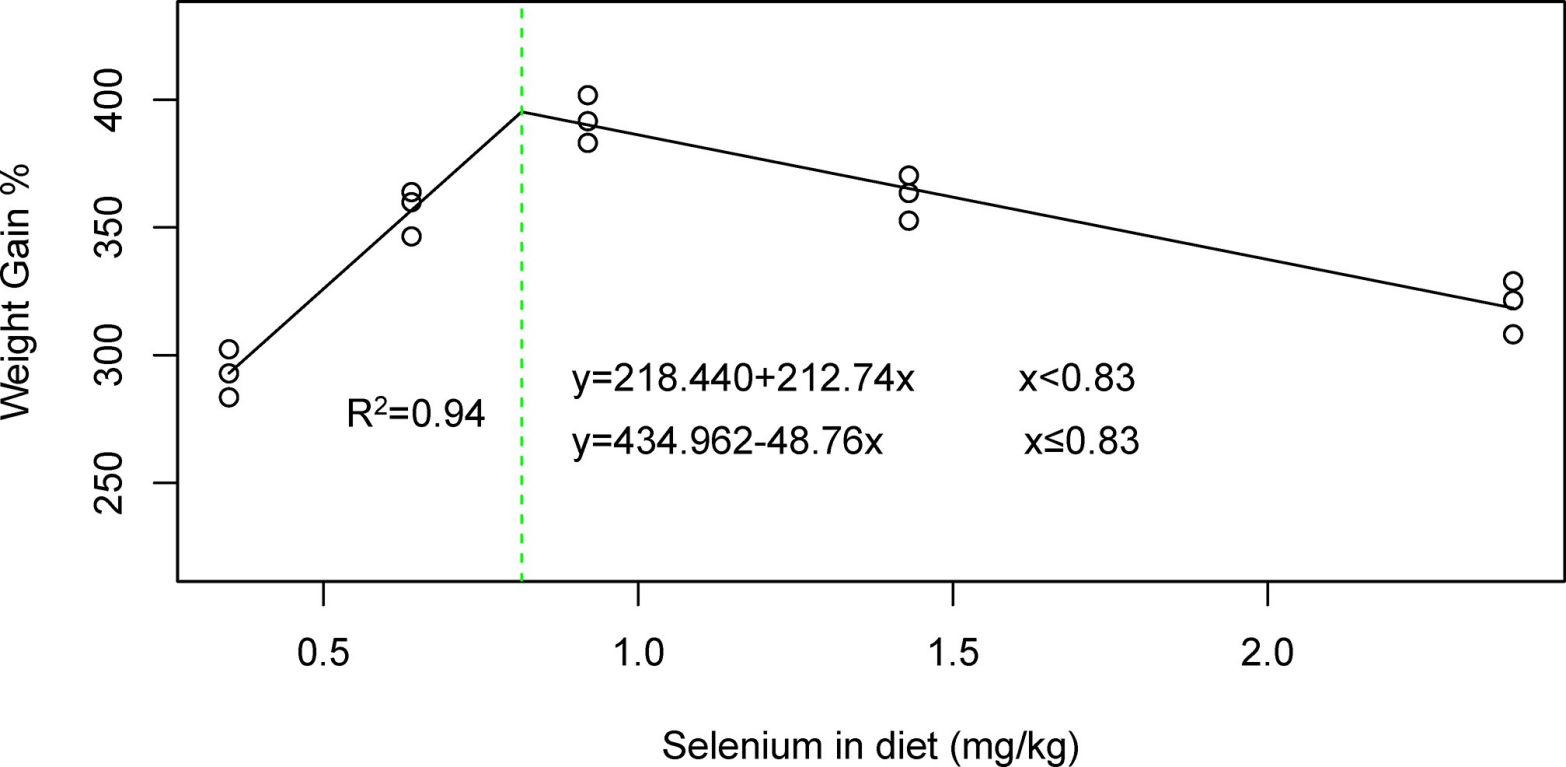
Broken-line regression analysis based on WG% data showing the optimal value of Se (mg/kg) for *C*. *mrigala*.

The supplementation of Se up to 0.92 mg/kg significantly increased (p<0.05) FBW, WG, WG%, and SGR by 25%, 34%, 33%, and 16%, respectively, compared with the control diet. However, a slight decline (p<0.05) in these parameters was noted with a further increase in Se concentration (Table 2). The best values (p<0.05) of FI and FCR were recorded at the 0.92 mg/kg Se level. No mortality was recorded throughout the trial. Based on broken-line regression analysis, supplementation with 0.83 mg/kg Se was recommended for the maximum WG% of *C*. *mrigala* (Fig 1).

**Table 2:** Growth performance and feed utilization of *Cirrhinus mrigala* given experimental diets.

Parameters	Se in diets (mg/kg)					p-values
	0.35	0.64	0.92	1.43	2.39	
**Initial body weight (g)**	7.44±0.13	7.42±0.09	7.48±0.09	7.42±0.13	7.44±0.13	0.974
**Final body weight (g)**	29.25±0.19^e^	33.88±0.24^c^	36.80±0.25^a^	34.31±0.06^b^	31.21±0.23^d^	< 0.001
**Weight gain (g)**	21.80±0.32^d^	26.46±0.33^b^	29.32±0.34^a^	26.88±0.17^b^	23.77±0.36^c^	< 0.001
**Weight gain%**	292.90±9.40^d^	356.72±9.08^b^	392.14±9.34^a^	362.13±8.91^b^	319.51±10.51^c^	< 0.001
**Feed intake (g fish** ^**-1**^ **)**	30.46±0.30^c^	30.76±0.40^bc^	31.33±0.51^b^	32.39±0.44^a^	30.46±0.40^c^	0.001
**Feed conversion ratio (g g** ^**-1**^ **)**	1.39±0.00^a^	1.16±0.00^d^	1.06±0.00^e^	1.20±0.00^c^	1.28±0.00^b^	< 0.001
**Specific growth rate (% day** ^**-1**^ **)**	1.52±0.02^d^	1.68± 0.02^b^	1.77±0.02^a^	1.70±0.02^b^	1.59±0.02^c^	< 0.001
**Survival rate%**	100	100	100	100	100	-

Data are shown as the mean ± SD of triplicate tanks. Means having dissimilar letters within a row indicate significant difference (p < 0.05).

### Carcass composition

Supplementation with Se significantly improved (p<0.05) the whole-body crude protein and crude fat while reducing (p<0.05) the moisture content of *C*. *mrigala*. However, crude ash remained unaffected by Se supplementation (Table 3). Fish fed the diets with 0.92 mg/kg and 1.43 mg/kg Se had significantly higher (p<0.05) whole-body crude protein, followed by the fish fed 2.39 mg/kg Se, then 0.64 mg/kg Se and finally the control diet. The crude fat content increased (p<0.05) from 4.30% to 5.33% with increasing Se supplementation levels up to 0.92 mg/kg and decreased (p<0.05) thereafter.

**Table 3:** Carcass composition analysis of *Cirrhinus mrigala* given experimental diets.

Parameters (%)	Se in diets (mg/kg)					p-values
	0.35	0.64	0.92	1.43	2.39	
**Moisture**	75.75±0.12^a^	74.32±0.14^b^	74.23±0.10^b^	74.42±0.13^b^	74.40±0.13^b^	<0.001
**Crude Protein**	16.56±0.12^d^	16.87±0.07^c^	17.84±0.08^a^	17.79±0.07^a^	17.12±0.10^b^	<0.001
**Crude Fat**	4.30±0.04^d^	4.68±0.06^c^	5.33±0.05^a^	5.04±0.05^b^	4.95±0.07^b^	<0.001
**Crude Ash**	2.48±0.13	2.41±0.08	2.45±0.10	2.41±0.09	2.47±0.12	0.918

Data are shown as the mean ± SD of triplicate tanks. Means with dissimilar letters within a row indicate significant differences (p < 0.05).

### Liver and serum antioxidant status and lipid peroxidation

The antioxidant response of *C*. *mrigala* in response to Se supplementation is summarized in Table 4. SOD activities responded positively (p<0.05) with higher Se concentrations up to 0.92 mg/kg diet. However, a slight decline (p<0.05) in its values was observed thereafter. CAT activities tended to increase (p<0.05) from 70.52 to 75.45 U/g prot up to the 0.92 mg/kg Se level and decreased significantly (p<0.05) beyond this level. Similarly, GPx activities improved (p<0.05) with Se levels, peaking at 0.92 mg/kg and declining (p<0.05) thereafter. MDA contents were reduced (p<0.05) from 3.67 to 3.11 mg/g prot with higher Se up to 0.92 mg/kg diet. However, a gradual increase in its content was recorded at higher Se levels.

**Table 4:** Antioxidant status and lipid peroxidation (MDA content) of *Cirrhinus mrigala* given experimental diets.

Parameters	Se in diets (mg/kg)					p-values
	0.35	0.64	0.92	1.43	2.39	
**Liver**						
**SOD (U/mg prot)**	6.56±0.20^d^	7.25±0.28^c^	8.34±0.18^a^	7.89±0.18^b^	7.84±0.25^b^	< 0.001
**CAT (U/mg prot)**	70.52±0.54^c^	72.70±1.32^b^	75.45±0.30^a^	73.22±0.68^b^	73.84±0.43^b^	< 0.001
**GPx (mU/mg prot)**	95.91±0.59^c^	96.72±0.47^c^	105.46±0.24^a^	99.16±0.73^b^	99.13±0.71^b^	< 0.001
**MDA (mg/g prot)**	3.67±0.04^a^	3.42 ± 0.04^b^	3.11±0.08^d^	3.27±0.03^c^	3.36±0.07^bc^	< 0.001
**Serum**						
**SOD (U/mg prot)**	6.35±0.14^d^	7.17±0.13^c^	8.08±0.1^a^	7.55±0.14^b^	7.5±0.13^b^	< 0.001
**CAT (U/mg prot)**	68.32±0.10^d^	69.12±0.08^c^	73.67±0.11^a^	71.53±0.10^b^	71.48±0.13^b^	< 0.001
**GPx (mU/mg prot)**	92.39±0.17^d^	93.7±0.15^c^	98.73±0.16^a^	95.9±0.17^b^	95.77±0.16^b^	< 0.001
**MDA (mg/g prot)**	3.73±0.02^a^	3.48±0.04^b^	3.21±0.03^d^	3.34±0.02^c^	3.39±0.03^c^	< 0.001

Data are presented as the mean ± SD of triplicate tanks. Means with dissimilar letters within a row indicate significant differences (p < 0.05). SOD: Superoxide dismutase; CAT: Catalase; GPx: Glutathione peroxidase; MDA: Malondialdehyde.

### Nonspecific immune response

Fish fed the Se-containing diets showed significantly elevated (p<0.05) lysozyme activity compared with those fed the control diet (Table 5). Furthermore, immunoglobulin increased (p<0.05) from 0.74 to 1.02 g/g pro as Se increased from 0.35 to 0.92 mg/kg diet and then declined significantly at the 1.43 and 2.39 mg/kg levels.

**Table 5:** Nonspecific immune parameters of *Cirrhinus mrigala* given experimental diets.

Parameters	Se in diets (mg/kg)					p-values
	0.35	0.64	0.92	1.43	2.39	
**Lysozyme activity (U/ml)**	9.40±0.13^b^	10.35±0.11^a^	10.56±0.12^a^	10.45±0.11^a^	10.43±0.11^a^	< 0.001
**Total immunoglobulin (g/g prot)**	0.74±0.01^d^	0.78±0.02^d^	1.02±0.03^a^	0.92±0.02^b^	0.85±0.02^c^	< 0.001

Data are shown as the mean ± SD of triplicate tanks. Means with dissimilar letters indicate significant differences (p < 0.05).

### Serum hepatic enzymes

Fish fed the diets containing ≤ 1.43 Se exhibited a nonsignificant difference in ALT values compared to the fish given the control diet. However, the highest values (p<0.05) of ALT were observed at the 2.39 mg/kg level. Similarly, a subsequent increase (p<0.05) in AST values was recorded as Se increased from 0.92 to 2.39 mg/kg diet. However, ALP remained unaffected by dietary treatment (Table 6).

**Table 6:** Hepatic enzyme activity of *Cirrhinus mrigala* given experimental diets.

Parameters (U/L)	Se in diets (mg/kg)					p-values
	0.35	0.64	0.92	1.43	2.39	
**Alkaline phosphatase**	87.09 ± 2.10	86.61 ± 2.30	88.47 ± 2.58	89.92 ± 2.55	91.51 ± 2.80	0.171
**Alanine aminotransferase**	25.30 ± 1.15^b^	24.80 ± 1.10^b^	25.50 ± 1.30^b^	26.64 ± 1.03^b^	29.50 ± 1.09^a^	0.003
**Aspartate aminotransferase**	11.22 ± 0.49^b^	10.98 ± 0.59^b^	10.96 ± 0.63^b^	13.33 ± 0.54^a^	14.22 ± 0.59^a^	< 0.001

Data are shown as the mean ± SD of triplicate tanks. Means with dissimilar letters within a row indicate significant differences (p < 0.05).

## Discussion

Feed is the most important component of aquaculture which consists of macro and micro-nutrients that are essential for the optimum growth performance and health status of fish [31]. Selenium is an essential mineral which plays various physiological functions in fish [32]. Furthermore, optimum level of Se is also important as under or over supplementation results in depressed growth performance or various physiological dysfunctions [32]. Fish require about 0.2–12 mg/kg Se in diet depending on various factors such as specie, size, feed formulation and culture conditions [10,16,33–39]. In this study, 0.83 mg/kg Se was calculated as optimum requirement of *C*. *mrigala* based on WG% (Fig 1). In this study, dietary supplementation of Se-NPs enhanced the growth performance and feed utilization in *C*. *mrigala*. Currenlty, no study has reported the optimum dietary Se-NPs for *C*. *mrigala*. Se has been shown to enhance growth hormone (thyroid) production in fish, contributing to improved growth performance [40]. Se interacts with the deiodinase enzyme, a key component in the regulation of thyroid hormones [41]. In fish, as in other vertebrates, the pituitary gland plays a crucial role in secreting thyroid hormones, which subsequently stimulate the production of growth hormones [42]. Moreover, Se enhances intracellular protein content in the epithelial cells of the intestine, which in turn improves the feed utilization and growth of fish [43]. Furthermore, the functional role of Se as a coenzyme in the synthesis of digestive enzymes improves the digestibility and absorption of nutrients in the intestine, resulting in better growth performance of fish [44].

The proximate composition of fish, encompassing parameters such as moisture, protein, lipid, and ash content, is of paramount importance in multiple contexts. It serves as a critical metric for assessing the nutritional value of fish [45]. In the current study, supplementation of Se-NPs significantly enhanced crude protein and crude lipid content in whole body of *C*. *mrigala* which coincides with previous studies [17,35,39,46]. This increase in protein and lipids in whole body can be explained as Se enhances the secretion of digestive enzymes which boost the digestion of proteins and lipids [44]. Furthermore, improvement in intestine health ultimate results in better absorption of nutrients which are stored in body [43]. Furthermore, Dietary Se participates in building selenoproteins that enhance proteins’ synthesis in the GIT and enhance protein accumulation in the tissues.

Aquaculture, especially intensive culture system, is prone to various stressors which lead oxidative stress which ultimately reduce growth performance and negatively impact the health status of fish [5]. During stress condition, free radicals are produced and fish have a natural antioxidant mechanism which neutralizes these free radicals [47]. However, when the production of free radicals exceeds the neutralizing capacity of antioxidant system, then these free radicals attack the lipids in cell membranes and produce malondialdehyde [43]. The main components of antioxidant defence system are catalase, superoxide dismutase and glutathione peroxidase which scavange free radicals. In current study, dietary supplementation of Se-NPs enhanced the antioxidant potential in *C*. *mrigala* which coincides with the previous studies [16,33,35–37,48]. This can be explained as Se has a key role in synthesis of glutathione peroxidase enzyme. This enzyme with the help of CAT and SOD neutralizes the free radicals ultimately protecting fish from deleterious effects of free radicals. Furthermore, Se-NPs have also been reported to enhance the gene expression of GPx [42].

Immunoglobulins are vital components of the adaptive immune system in fish, serving key roles in pathogen defense and immune regulation [49]. They are primarily involved in the recognition and neutralization of specific antigens, thereby preventing infections and contributing to the organism’s overall immune response [49,50]. Upon antigen binding, immunoglobulins can activate complement proteins and phagocytic cells, which work to eliminate pathogens from the host [50]. It was observed that the serum immunoglobulin of *C*. *mrigala* increased significantly with Se-NP supplementation which is in line with previous studies [35,40]. This might be due to stimulation of GPx in those cells, which in turn activates and protects B lymphocytes, resulting in a better immune response [51].

Lysozymes complement the function of immunoglobulins in fish immune defense. These enzymes are part of the innate immune system and are known for their ability to break down bacterial cell walls by hydrolyzing the peptidoglycan layer [52]. This bacteriolytic activity is crucial for protecting fish against bacterial infections, particularly in environments where they are exposed to a wide variety of microbial threats. Lysozymes are found in various tissues and body fluids of fish, including mucus, blood, and internal organs, providing a broad-spectrum antimicrobial barrier [53]. Supplementation with Se had a significant positive impact on lysozyme activities which is in accordance to previous studies [22,36,37,40]. This enhancement in lysozyme activity might be due to well-known immunomodulatory role of Se in the immune system via contributing to the proliferation, differentiation, and regulation of the immune cells (e.g., lymphocytes and neutrophils) [54].

ALP, ALT, and AST are important enzymes involved in metabolism. A significant increase in ALT and AST levels was recorded at the 2.39 mg/kg level in this study, indicating that an overdose of Se may cause toxic effects on the liver health of fish. It was reported that Se mainly accumulates in the liver of fish, and a high concentration of Se may lead to the degeneration of liver tissues [55]. Similarly, Ashouri, Keyvanshokooh [16] noted the highest levels of serum ALT and AST at the 2 mg/kg Se-NP level in common carp. In contrast, Yu, Zhang [56] reported a decreasing trend in AST and ALT values with increasing levels of Se-NPs in *Ctenopharyngodon* idella, whereas no significant effects of Se-NPs on AST and ALT levels were observed in *Dicentrarchus labrax* [22]. The improvements in hepatic enzymes activities with Se supplementation can be associated with the enhancement of antioxidant status, immune response and overall health of fish as described previously.

## Conclusion

The findings of the study showed that supplementation with up to 0.92 mg/kg Se in the form of NPs significantly improved the growth performance, feed utilization, whole-body composition, antioxidant capacity, and immunity of *C*. *mrigala*. Based on broken-line regression analysis, 0.83 mg/kg Se is recommended for the optimum performance of *C*. *mrigala*. To date, very few studies have investigated the effect of Se-NPs on carp. Therefore, more concerning efforts should be devoted to this regard.

## References

[1] Boyd CE, D’Abramo LR, Glencross BD, Huyben DC, Juarez LM, Lockwood GS, et al. Achieving sustainable aquaculture: Historical and current perspectives and future needs and challenges. J World Aquacult Soc. 2020;51(3):578–633. doi: 10.1111/jwas.12714

[2] Martos-Sitcha JA, Mancera JM, Prunet P, Magnoni LJ. Editorial: Welfare and Stressors in Fish: Challenges Facing Aquaculture. Front Physiol. 2020;11:162. Epub 20200225. doi: 10.3389/fphys.2020.00162 ; PubMed Central PMCID: PMC7055460. 32174844 PMC7055460

[3] Tort L. Stress and immune modulation in fish. Developmental & Comparative Immunology. 2011;35(12):1366–75. doi: 10.1016/j.dci.2011.07.002 21782845

[4] Cabello FC. Heavy use of prophylactic antibiotics in aquaculture: a growing problem for human and animal health and for the environment. Environmental Microbiology. 2006;8(7):1137–44. doi: 10.1111/j.1462-2920.2006.01054.x 16817922

[5] Ciji A, Akhtar MS. Stress management in aquaculture: a review of dietary interventions. Rev Aquac. 2021;13(4):2190–247. doi: 10.1111/raq.12565

[6] Raymond LJ, Ralston NVC. Selenium’s importance in regulatory issues regarding mercury. Fuel Processing Technology. 2009;90(11):1333–8. doi: 10.1016/j.fuproc.2009.07.012

[7] Khan KU, Zuberi A, Nazir S, Ullah I, Jamil Z, Sarwar H. Synergistic effects of dietary nano selenium and vitamin C on growth, feeding, and physiological parameters of mahseer fish (Tor putitora). Aquac Rep. 2017;5:70–5. doi: 10.1016/j.aqrep.2017.01.002

[8] Antony Jesu Prabhu P, Schrama JW, Kaushik SJ. Mineral requirements of fish: a systematic review. Reviews in Aquaculture. 2016;8(2):172–219. doi: 10.1111/raq.12090

[9] Poppe TT, Hastein T, Froslie A, Koppgang N, Nordheim G. Nutritional aspects of Haemorrhagic Syndrome (’Hitra Disease’) in farmed Atlantic salmon Salmo salar. Diseases of Aquatic Organisms. 1985;1:155–62. doi: 10.3354/dao001155

[10] Han D, Xie S, Liu M, Xiao X, Liu H, Zhu X, et al. The effects of dietary selenium on growth performances, oxidative stress and tissue selenium concentration of gibel carp (Carassius auratus gibelio). Aquaculture Nutrition. 2011;17(3):e741–e9. doi: 10.1111/j.1365-2095.2010.00841.x

[11] Kouba A, Velíšek J, Stará A, Masojídek J, Kozák P. Supplementation with Sodium Selenite and Selenium-Enriched Microalgae Biomass Show Varying Effects on Blood Enzymes Activities, Antioxidant Response, and Accumulation in Common Barbel (Barbus barbus). BioMed Research International. 2014;2014:1–8. doi: 10.1155/2014/408270 24772422 PMC3955621

[12] Sarkar B, Bhattacharjee S, Daware A, Tribedi P, Krishnani KK, Minhas PS. Selenium Nanoparticles for Stress-Resilient Fish and Livestock. Nanoscale Research Letters. 2015;10(1). doi: 10.1186/s11671-015-1073-2 26400834 PMC4580674

[13] Saffari S, Keyvanshokooh S, Zakeri M, Johari SA, Pasha-Zanoosi H, Mozanzadeh MT. Effects of dietary organic, inorganic, and nanoparticulate selenium sources on growth, hemato-immunological, and serum biochemical parameters of common carp (Cyprinus carpio). Fish Physiology and Biochemistry. 2018;44(4):1087–97. doi: 10.1007/s10695-018-0496-y 29663181

[14] Wang H, Zhang J, Yu H. Elemental selenium at nano size possesses lower toxicity without compromising the fundamental effect on selenoenzymes: Comparison with selenomethionine in mice. Free Radical Biology and Medicine. 2007;42(10):1524–33. doi: 10.1016/j.freeradbiomed.2007.02.013 17448899

[15] Zhou X, Wang Y, Gu Q, Li W. Effects of different dietary selenium sources (selenium nanoparticle and selenomethionine) on growth performance, muscle composition and glutathione peroxidase enzyme activity of crucian carp (Carassius auratus gibelio). Aquaculture. 2009;291(1–2):78–81. doi: 10.1016/j.aquaculture.2009.03.007

[16] Ashouri S, Keyvanshokooh S, Salati AP, Johari SA, Pasha-Zanoosi H. Effects of different levels of dietary selenium nanoparticles on growth performance, muscle composition, blood biochemical profiles and antioxidant status of common carp (Cyprinus carpio). Aquaculture. 2015;446:25–9. doi: 10.1016/j.aquaculture.2015.04.021

[17] Dawood MAO, Koshio S, Zaineldin AI, Van Doan H, Ahmed HA, Elsabagh M, et al. An evaluation of dietary selenium nanoparticles for red sea bream (Pagrus major) aquaculture: growth, tissue bioaccumulation, and antioxidative responses. Environmental Science and Pollution Research. 2019;26(30):30876–84. doi: 10.1007/s11356-019-06223-6 31446600

[18] Khizar A, Fatima M, Shah SZH, Khan N, Maryam, Khan F. Optimum dietary calcium requirement of Hypophthalmichthys molitrix juveniles. Aquaculture Research. 2022;53(16):5455–62. doi: 10.1111/are.16026

[19] Berka R. The transport of live fish: a review: Food and Agriculture Organization of the United Nations Rome, Italy; 1986.

[20] Ali W, Fatima M, Shah SZH, Khan N, Naveed S. Star anise extract supplementation improved growth performance, hepatic-antioxidant enzyme status, hematology, serum biochemistry, and survival against crowding stress in Catla catla. Aquaculture. 2024;584. doi: 10.1016/j.aquaculture.2024.740674

[21] Fatima M, Afzal M, Shah SZH. Effect of dietary oxidized oil and vitamin E on growth performance, lipid peroxidation and fatty acid profile ofLabeo rohitafingerlings. Aquac Nutr. 2019;25(2):281–91. doi: 10.1111/anu.12851

[22] Abd El-Kader MF, Fath El-Bab AF, Abd-Elghany MF, Abdel-Warith AA, Younis EM, Dawood MAO. Selenium Nanoparticles Act Potentially on the Growth Performance, Hemato-Biochemical Indices, Antioxidative, and Immune-Related Genes of European Seabass (Dicentrarchus labrax). Biological Trace Element Ressearch. 2021;199(8):3126–34. Epub 20201015. doi: 10.1007/s12011-020-02431-1 . 33058040

[23] AOAC. Official methods of analysis of AOAC International. Twentieth edition. ed. Rockville, MD: AOAC International; 2016. 2 volumes: illustrations p.

[24] Mushtaq M, Fatima M, Shah SZH, Khan N, Naveed S, Khan M. Effects of sodium selenite, selenium methionine, and selenium yeast on growth performance, carcass composition, blood biochemistry, and antioxidant status of intensively reared Hypophthalmichthys molitrix. Aquac Rep. 2022;24. doi: 10.1016/j.aqrep.2022.101182 PMC9480980 36112655

[25] Civello PM, Martinez GA, Chaves AR, Anon MC. Peroxidase from Strawberry Fruit (Fragaria ananassa Duch.): Partial Purification and Determination of Some Properties. Journal of Agricultural and Food Chemistry. 2002;43(10):2596–601. doi: 10.1021/jf00058a008

[26] Chance B, Maehly AC. [136] Assay of catalases and peroxidases. Methods in Enzymology 1955. p. 764–75. 10.1002/9780470110171.ch14 13193536

[27] Giannopolitis CN, Ries SK. Superoxide dismutases: I. Occurrence in higher plants. Plant Physiol. 1977;59(2):309–14. doi: 10.1104/pp.59.2.309 ; PubMed Central PMCID: PMC542387. 16659839 PMC542387

[28] Gatta Pirini, Testi Vignola, Monetti. The influence of different levels of dietary vitamin E on sea bassDicentrarchus labraxflesh quality. Aquac Nutr. 2000;6(1):47–52. doi: 10.1046/j.1365-2095.2000.00127.x

[29] Ellis A. Lysozyme Assays. Stolen JS FT, Anderson DP, Roberson BS, Van Muiswinkel WB, editor: NJ: SOS Publications; 1990. 101–3 p.

[30] Anderson DP, Siwicki AK. Basic hematology and serology for fish health programs. Manila, Phillipines: Fish Health Section, Asian Fisheries Society; 1995. 18 p.

[31] Tacon AGJ, Metian M. Feed Matters: Satisfying the Feed Demand of Aquaculture. Reviews in Fisheries Science & Aquaculture. 2015;23(1):1–10. doi: 10.1080/23308249.2014.987209

[32] Oliva‐Teles A. Nutrition and health of aquaculture fish. Journal of Fish Diseases. 2012;35(2):83–108. doi: 10.1111/j.1365-2761.2011.01333.x 22233511

[33] Hao X, Ling Q, Hong F. Effects of dietary selenium on the pathological changes and oxidative stress in loach (Paramisgurnus dabryanus). Fish Physiology and Biochemistry. 2014;40(5):1313–23. doi: 10.1007/s10695-014-9926-7 24633928

[34] Abd El-Kader MF, Fath El-Bab AF, Shoukry M, Abdel-Warith A-WA, Younis EM, Moustafa EM, et al. Evaluating the possible feeding strategies of selenium nanoparticles on the growth rate and wellbeing of European seabass (Dicentrarchus labrax). Aquac Rep. 2020;18. doi: 10.1016/j.aqrep.2020.100539

[35] Sultan I, Fatima M, Shah SZH, Khan N, Nadeem H, Ali W. Effects of dietary nano-selenium concentration on growth, proximate chemical composition and antioxidant enzymes, and physiological responses to stressors by juvenile Catla catla. Aquaculture. 2024;588. doi: 10.1016/j.aquaculture.2024.740914

[36] Abdollahi-Mousavi SE, Keyvanshokooh S, Torfi Mozanzadeh M, Ghasemi A. Efficacy of nutritional selenium nanoparticles on growth performance, immune response, antioxidant capacity, expression of growth and immune-related genes, and post-stress recovery in juvenile Sobaity seabream (Sparidentex hasta). Fish & Shellfish Immunology. 2024;147. doi: 10.1016/j.fsi.2024.109452 38360194

[37] Eissa E-SH, Bazina WK, Abd El-Aziz YM, Abd Elghany NA, Tawfik WA, Mossa MI, et al. Nano-selenium impacts on growth performance, digestive enzymes, antioxidant, immune resistance and histopathological scores of Nile tilapia, Oreochromis niloticus against Aspergillus flavus infection. Aquaculture International. 2023;32(2):1587–611. doi: 10.1007/s10499-023-01230-4

[38] Dawit Moges F, Hamdi H, Al-Barty A, Zaid AA, Sundaray M, Parashar SKS, et al. Effects of selenium nanoparticle on the growth performance and nutritional quality in Nile Tilapia, Oreochromis niloticus. PLoS One. 2022;17(6):e0268348. Epub 2022/06/03. doi: 10.1371/journal.pone.0268348 ; PubMed Central PMCID: PMC9162325. 35653406 PMC9162325

[39] Senthamara MD, Rekha M, Rajan MR. Incorporation of Nano Selenium in Fish Diet and Assessment of Growth Performance and Biochemical Criteria of Labeo rohita. Journal of Environmental Nanotechnology. 2024;13(1):01–9. doi: 10.13074/jent.2024.03.234490

[40] Rathore SS, Murthy HS, Mamun MA-A, Nasren S, Rakesh K, Kumar BTN, et al. Nano-selenium Supplementation to Ameliorate Nutrition Physiology, Immune Response, Antioxidant System and Disease Resistance Against Aeromonas hydrophila in Monosex Nile Tilapia (Oreochromis niloticus). Biological Trace Element Research. 2020;199(8):3073–88. doi: 10.1007/s12011-020-02416-0 33025518

[41] Holben DH, Smith AM. The Diverse Role of Selenium within Selenoproteins. Journal of the American Dietetic Association. 1999;99(7):836–43. doi: 10.1016/s0002-8223(99)00198-4 10405682

[42] Mehdi Y, Hornick J-L, Istasse L, Dufrasne I. Selenium in the Environment, Metabolism and Involvement in Body Functions. Molecules. 2013;18(3):3292–311. doi: 10.3390/molecules18033292 23486107 PMC6270138

[43] Saffari S, Keyvanshokooh S, Zakeri M, Johari SA, Pasha-Zanoosi H. Effects of different dietary selenium sources (sodium selenite, selenomethionine and nanoselenium) on growth performance, muscle composition, blood enzymes and antioxidant status of common carp (Cyprinus carpio). Aquac Nutr. 2017;23(3):611–7. doi: 10.1111/anu.12428

[44] Shi L, Xun W, Yue W, Zhang C, Ren Y, Liu Q, et al. Effect of elemental nano-selenium on feed digestibility, rumen fermentation, and purine derivatives in sheep. Animal Feed Science and Technology. 2011;163(2–4):136–42. doi: 10.1016/j.anifeedsci.2010.10.016

[45] Mohanty BP, Mahanty A, Ganguly S, Mitra T, Karunakaran D, Anandan R. Nutritional composition of food fishes and their importance in providing food and nutritional security. Food Chemistry. 2019;293:561–70. doi: 10.1016/j.foodchem.2017.11.039 31151648

[46] Dawood MAO, Dawit Moges F, Hamdi H, Al-Barty A, Zaid AA, Sundaray M, et al. Effects of selenium nanoparticle on the growth performance and nutritional quality in Nile Tilapia, Oreochromis niloticus. Plos One. 2022;17(6). doi: 10.1371/journal.pone.0268348 35653406 PMC9162325

[47] Chowdhury S, Saikia S. Oxidative Stress in Fish: A Review. Journal of Scientific Research. 2020;12(1).

[48] Dawood MAO, Zommara M, Eweedah NM, Helal AI, Aboel-Darag MA. The potential role of nano-selenium and vitamin C on the performances of Nile tilapia (Oreochromis niloticus). Environmental Science and Pollution Research. 2020;27(9):9843–52. doi: 10.1007/s11356-020-07651-5 31925699

[49] Mashoof S, Criscitiello M. Fish Immunoglobulins. Biology. 2016;5(4). doi: 10.3390/biology5040045 27879632 PMC5192425

[50] Yu Y, Wang Q, Huang Z, Ding L, Xu Z. Immunoglobulins, Mucosal Immunity and Vaccination in Teleost Fish. Frontiers in Immunology. 2020;11. doi: 10.3389/fimmu.2020.567941 33123139 PMC7566178

[51] Combs GF, Combs SB. 9—SELENIUM IN IMMUNITY AND INFECTION. In: Combs GF, Combs SB, editors. The Role of Selenium in Nutrition: Academic Press; 1986. p. 401–11.

[52] Song Q, Xiao Y, Xiao Z, Liu T, Li J, Li P, et al. Lysozymes in Fish. Journal of Agricultural and Food Chemistry. 2021;69(50):15039–51. doi: 10.1021/acs.jafc.1c06676 34890178

[53] Saurabh S, Sahoo PK. Lysozyme: an important defence molecule of fish innate immune system. Aquaculture Research. 2008;39(3):223–39. doi: 10.1111/j.1365-2109.2007.01883.x

[54] Wang L, Sagada G, Wang R, Li P, Xu B, Zhang C, et al. Different forms of selenium supplementation in fish feed: The bioavailability, nutritional functions, and potential toxicity. Aquaculture. 2022;549. doi: 10.1016/j.aquaculture.2021.737819

[55] Sorensen EMB, Cumbie PM, Bauer TL, Bell JS, Harlan CW. Histopathological, hematological, condition-factor, and organ weight changes associated with selenium accumulation in fish from Belews Lake, North Carolina. Arch Environ Contam Toxicol. 1984;13(2):153–62. doi: 10.1007/BF01055872 6721580

[56] Yu H, Zhang C, Zhang X, Wang C, Li P, Liu G, et al. Dietary nano‐selenium enhances antioxidant capacity and hypoxia tolerance of grass carp Ctenopharyngodon idella fed with high‐fat diet. Aquaculture Nutrition. 2019;26(2):545–57. doi: 10.1111/anu.13016

